# Sarcopenic Obesity in Community-Dwelling Spanish Adults Older than 65 Years

**DOI:** 10.3390/nu15234932

**Published:** 2023-11-27

**Authors:** Angela Diago-Galmés, Carlos Guillamon-Escudero, Jose M. Tenías-Burillo, Jose M. Soriano, Julio Fernández-Garrido

**Affiliations:** 1 Hospital Universitario de La Plana, 12540 Castellón, Spain; angela94dg@gmail.com; 2 Hospital General Universitari de Castelló, 12004 Castellón, Spain; carlos_ge@hotmail.es; 3 Department of Preventive Medicine, Hospital Pare Jofré, 46017 Valencia, Spain; tenias_jma@gva.es; 4 Food & Health Lab, Institute of Materials Science, University of Valencia, 43617 Valencia, Spain; jose.soriano@uv.es; 5 Joint Research Unit on Endocrinology, Nutrition and Clinical Dietetics, University of Valencia-Health Research Institute La Fe, 46026 Valencia, Spain; 6 Department of Nursing, Faculty of Nursing and Podiatry, University of Valencia, 46010 Valencia, Spain

**Keywords:** sarcopenic obesity, older adults, muscle mass, adipose tissue, diagnostic criteria

## Abstract

Sarcopenic obesity (SO) is diagnosed when sarcopenia and obesity coexist in patients. The objective of this study was to determine the prevalence of SO under different diagnostic criteria in community-dwelling Spanish adults aged over 65 years residing in Valencia (Spain). The research was conducted as an observational and cross-sectional study with a sample size of 202 subjects. To diagnose sarcopenia, we used the tests proposed by the European Working Group on Sarcopenia in Older People in 2019 (EWGSOP2): SARC-F, grip strength, sit-to-stand, gait speed, Appendicular Skeletal Muscle Mass (ASMM), and Short Physical Performance Battery (SPPB). For obesity diagnosis, we used body mass index (BMI), waist circumference (WC), total body fat percentage (%TBF), and tricipital skinfold (TS). The prevalence of SO was 16.5% in women and 29% in men, according to any of the diagnostic criteria used to determine obesity. A higher proportion of SO was observed as age increased in both groups, although no significant differences were found. Most values obtained in tests related to SO diagnosis were worse in the group affected by the disease; however, there were two exceptions related to the amount of ASMM. In total, 18.8% of the participants presented SO according to any diagnostic criteria related to obesity. Our results suggest significant differences in the number of SO cases depending on diagnostic criteria used to determine the participants’ obesity. BMI, WC, and TBF% were shown as principal variables to be included in obesity diagnosis within the SO construct. These findings underscore the need to unify criteria to standardize the diagnosis of SO in the global population.

## 1. Introduction

During the last decade, the clinical definition of sarcopenia has been modified several times due to the development of muscle tissue physiology knowledge and the understanding of its relationship with the symptoms presented by patients. Presently, it is defined as the loss of strength, muscle mass, and physical performance associated with aging [1,2]. During this period, a noticeable decline in muscle mass has been observed, leading to an increase in the percentage of total body fat mass [3], potentially contributing to overweight and obesity in older adults, either individually or collectively.

The coexistence of sarcopenia and obesity is prevalent in this population group, giving rise to the term “sarcopenic obesity” (SO). SO encompasses individuals experiencing both sarcopenia and obesity, accompanied by various associated metabolic issues [4,5,6]. The term has a relatively short historical trajectory, with its first mention in the scientific literature dating back to 1996 in a study by Heber et al. [7]. Consequently, there are divergent definitions of this pathology, contributing to varied results across multiple studies [5,7,8,9,10]. Baumgartner et al. [8] defined SO as the simultaneous presence of skeletal muscle mass two standard deviations below the mean for young people and a body fat percentage exceeding the median [11]. Subsequent studies [4,10,12,13] characterized SO as the two lowest quintiles of muscle mass and the two highest quintiles of fat mass. Kim et al. [10] introduced a new formula defining SO using Janssen’s definition [9] for sarcopenia and Davison’s definition [14] for obesity determination.

There is a lack of consensus on the criteria used for SO diagnosis [10]. This situation affected its prevalence estimation in older people, ranging from 3% to 18%, as evident in numerous studies [5,15].

The present study aims to establish the prevalence of SO in community-dwelling older adults residing in their own homes (not admitted to hospitals or nursing homes) in Valencia, Spain. This will be achieved by using and comparing different diagnostic methods for obesity and criteria proposed by EWGSOP2 [16] for sarcopenia diagnosis.

## 2. Materials and Methods

### 2.1. Participants (Study Population)

A descriptive cross-sectional study was conducted in community-dwelling older adults aged over 65 who participated in activities at a community center for older individuals in the neighborhood of Benimaclet, Valencia, Spain. The study aimed to exclude cases of sarcopenic obesity (SO) secondary to other pathologies that could potentially interfere with the analysis. Therefore, participants with a Barthel scale score [17] below 60 points were excluded, ensuring that all subjects exhibited independence or mild dependence in performing basic activities of daily living (BADL).

This research was carried out in collaboration with the Cathedra of Healthy, Active and Participative Aging established between the University of Valencia and the City Council of Valencia. Participation was voluntary, and none of the older individuals involved in this study were hospitalized during the research; all resided in their private homes.

The inclusion criteria for participants were: (a) age over 65; (b) registration as a user of the Benimaclet community center for older people in Valencia, Spain; (c) Barthel index equal to or greater than 60 points; and (d) completion of consent and ability to understand and complete all tests included in this study. The exclusion criteria were: (a) suffering from a disease that causes severe muscle mass impairment, to avoid the impact of secondary SO and (b) absence from the center on the days of this study due to forgetfulness, lack of time, or health problems.

**Figure 1:**
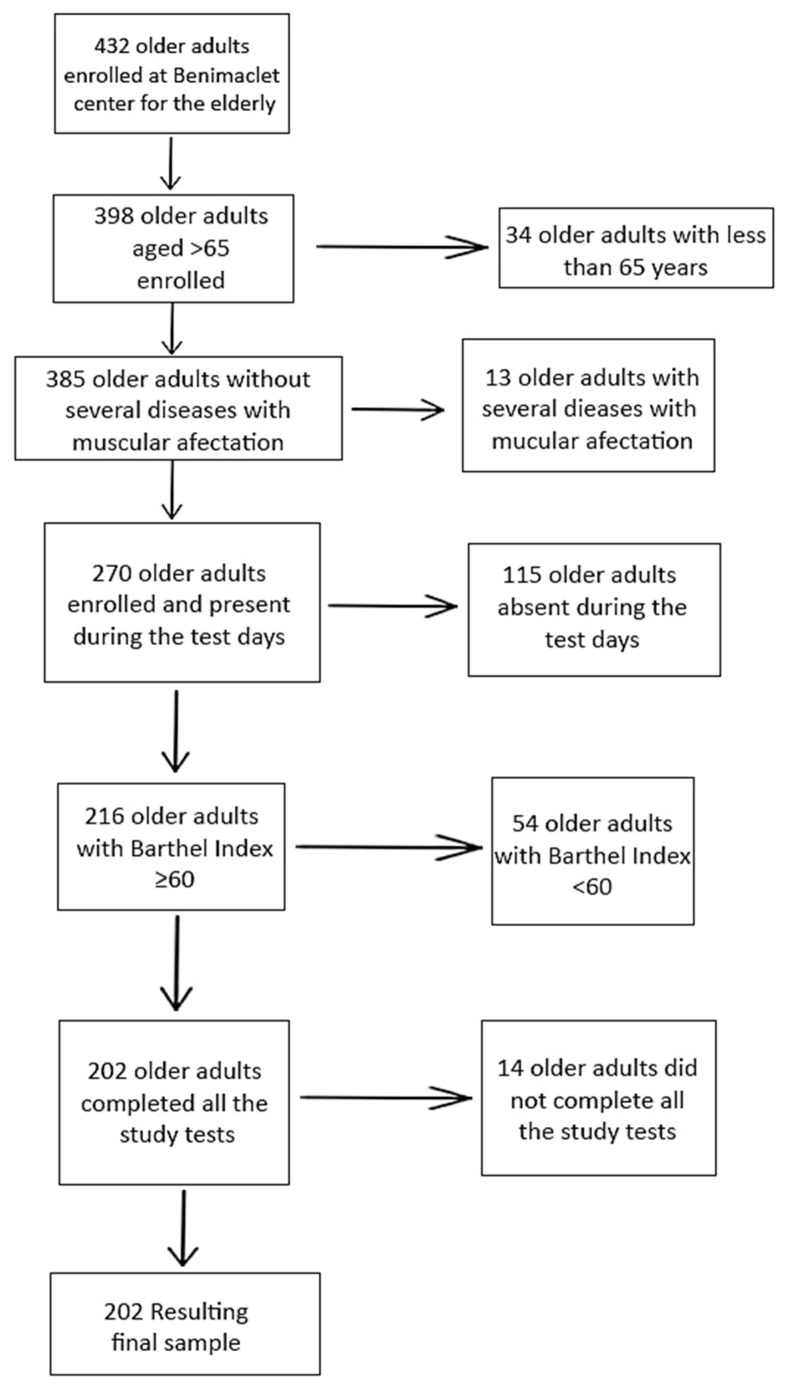
Recruitment of older adults for participation in this study.

The initial sample comprised 432 older people (both women and men), with 34 excluded for being under 65 years old. Subjects with diseases causing severe muscle mass impairment (*n* = 13), those not meeting the Barthel score criterion (*n* = 54), those absent on the study days (*n* = 115), and those not completing the study (*n* = 14) were excluded from the final sample. The participation percentage was 46.76%, resulting in a final sample size of 202 people (Figure 1).

### 2.2. Sample Size Estimation

After reviewing recent studies conducted by various authors [5,6,15] on the prevalence of SO in non-institutionalized older patients, it was observed that this variable ranged approximately from 3% to 18%, approximately. Given the variability in SO prevalence in community-dwelling populations reported in the literature, the authors opted to use the midpoint between the lowest and highest values (10.5%) as the initial point for calculating the sample size. Taking into consideration this assumed expected prevalence, the authors allowed for an estimation error ranging from 4% to 5% and a confidence interval of 95% during the sample size calculation. Consequently, it was determined that it would be necessary to include between 145 participants (in the case of lower prevalence and precision) and 226 subjects (in the case of higher prevalence and precision) to ensure a statistically stable and reliable sample.

A sample size exceeding two hundred cases, as observed in this study, is considered sufficient for estimating the prevalence of sarcopenic obesity (SO) with an acceptable level of precision. The calculations for sample size were conducted using EPIDAT 4.2 software.

### 2.3. Exam Protocol and Measurements

#### 2.3.1. General Information

Researchers collected general information from the participants using a custom questionnaire. The variables included were age and sex, the type of coexistence at home, and any diseases reported by the participants.

#### 2.3.2. Degree of Dependency

To assess the participants’ level of dependency, the Barthel index was used. This scale, validated for the Spanish population [17] and widely used in clinical settings [18,19,20], included participants with scores ranging from 60 (indicating mild dependency) to 100 (indicating total independence). This ensured that none of the participants faced significant difficulties in carrying out basic activities of daily living (BADL).

#### 2.3.3. Sarcopenia Screening

In addition to completing this study’s requirements, and solely to verify the reliability of the SARC-F questionnaire, all subjects completed it, regardless of whether there was suspicion of sarcopenia. The SARC-F questionnaire [21], recommended by the European Working Group on Sarcopenia in Older People (EWGSOP2) [16] for universal sarcopenia screening, comprises five sections assessing perceived strength, the use of walking devices, difficulty in getting up from a chair and climbing stairs, and the frequency of falls. A score equal to or less than four was considered indicative of susceptibility to sarcopenia, although it had no practical impact on subsequent diagnostic decisions.

#### 2.3.4. Diagnosis of Sarcopenic Pathology

The diagnosis of sarcopenic pathology was determined based on the latest recommendations from EWGSOP2 [16], which integrates three dimensions: low muscle strength [22], low muscle quantity or quality [23], and low physical performance [24]. These dimensions were individually analyzed using corresponding validated tools. Additionally, the SARC-F questionnaire was utilized to assess the ability to identify cases of sarcopenia [21].

Following the EWGSOP2 criteria [16], participants were classified into three stages of the disease: probable sarcopenia, confirmed sarcopenia, and severe sarcopenia. ‘Probable sarcopenia’ was assigned to subjects presenting only low muscle strength in the lower and upper body. When the evidence of the low quantity or quality of muscle mass accompanied this muscle strength loss, the participant was classified as having ‘confirmed sarcopenia’. Finally, if, in addition to these two conditions, subjects showed a decrease in physical performance, sarcopenia was classified as ‘severe sarcopenia’.

#### 2.3.5. Grip Strength (Upper Body)

Upper body strength was determined using manual ergometry [25,26,27,28,29] with a Jamar 5030J1 manual analog hydraulic dynamometer, featuring a measurement scale of 0–90 kg/force (kg/f) and a precision of ±2 kg.

The measurement protocol involved two attempts with each hand to achieve maximum voluntary grip strength, allowing a one-minute rest between attempts. The highest score obtained from the four total attempts was considered the final value. Measurements were taken with participants sitting in a chair with their backs straight, arms flexed at a 90-degree angle, and not resting on any surface. Indicative values for decreased strength were considered to be less than 27 kg in men and 16 kg in women.

#### 2.3.6. Lower Body Strength

Lower body strength was assessed using the “sit to stand” test, involving 5 repetitions for its practicality and simplicity. The test, proposed by EWGSOP2 [16], required participants to perform 5 chair squats at the maximum possible speed without using any manual support.

The result of the test was evaluated based on the time taken by participants, with values exceeding 15 s considered indicative of decreased strength, regardless of sex.

#### 2.3.7. Appendicular Skeletal Muscle Mass (ASMM)

ASMM, essential for categorizing sarcopenia cases, was determined using the equation proposed by Kyle et al. [30]. The formula utilized results obtained from bioelectrical impedance (BIA) performed with a digitally calibrated scale (TANITA DC 430mA-S^®^, Tokyo, Japan, accuracy ±0.05 kg). Values below 20 kg in men and 15 kg in women were considered indicative of a decrease (deficit) in muscle mass, following EWGSOP2 classification [16].

#### 2.3.8. Physical Performance

Physical was measured using the 4 m walking speed test [31,32] and the Short Physical Performance Battery test (SPPB test), following EWGSOP2 recommendations [16]. The 4 m walking speed test involved measuring the time it took participants to walk a distance of 4 m at their usual speed, with values below 0.8 m/s considered indicative of physical performance impairment.

The SPPB test included three assessments for balance, gait speed, and lower body strength [33,34]. The total result score was the sum of scores obtained in the three tests, with values equal to or less than 8 points, regardless of sex, considered indicative of physical performance deterioration.

#### 2.3.9. Diagnosis of Obesity

To determine the obesity of the participants, diagnostic criteria indicative of the pathology were utilized. The selection of these criteria was based on an analysis of the latest evidence on sarcopenic obesity (SO), revealing significant heterogeneity in the methodology related to determining this pathology [8,10,28,35].

#### 2.3.10. Body Mass Index (BMI)

The body mass index (BMI) of participants was analyzed using the standard formula (kg/m^2^). Individuals with a BMI greater than or equal to 30 (kg/m^2^) were classified as obese according to the World Health Organization (WHO) criteria [28].

#### 2.3.11. Waist Circumference (WC)

Waist circumference was measured following the criteria of the International Society for the Advancement of Kinanthropometry (ISAK) [36,37], conducted by technicians qualified for anthropometric measurements. A retractable, inextensible measuring tape was used. Participants with values greater than 88 cm in women and 102 cm in men were classified as obese [28].

#### 2.3.12. Total Body Fat Percentage (TBF%)

The percentage of total body fat was measured using bioimpedance analysis (BIA), following the latest recommendations to obtain precise results. Following Baumgartner’s criteria [11], individuals with a total body fat percentage greater than or equal to 38% were considered obese.

#### 2.3.13. Tricipital Skinfold (TS)

Triceps skinfold was assessed by qualified kinanthropometrists, as recommended by ISAK, using a Holtain caliper with an accuracy of ±0.2 mm. Values equal to or greater than the 85th percentile, based on the criteria presented by Kuczmarski et al. [37], were classified as an excess of body fat and, consequently, indicative of obesity.

#### 2.3.14. Diagnosis of Sarcopenic Obesity (SO)

To make SO diagnosis, various definitions were found that implied the use of certain diagnostic criteria related to obesity and sarcopenia. In this study, SO was diagnosed when obesity and sarcopenia coexisted in patients [28]. Thus, it was made by combining the EWGSOP2 criteria for sarcopenia and different diagnostic methods for obesity. The individualized use of each criterion related to obesity was performed with the aim of knowing SO prevalence as a function of each one of them. This methodology allows for the comparison between different prevalence of SO, taking into account that the acceptance of one or another diagnostic method for obesity is a relevant factor in terms of pathology detection.

**Figure 2:**
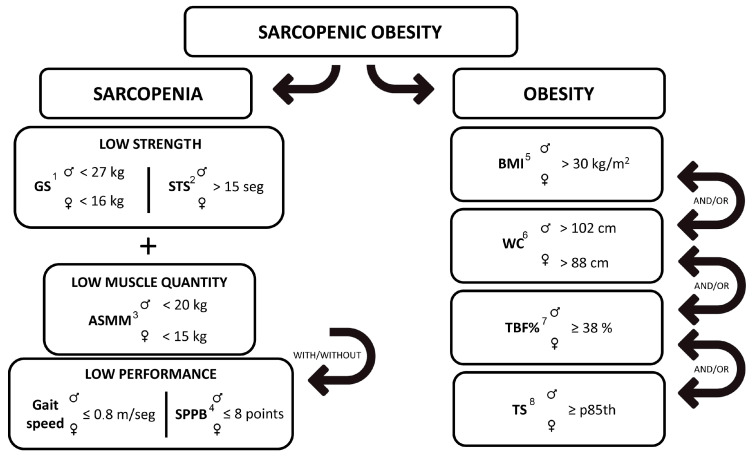
Sarcopenic obesity diagnosis algorithm. ^1^ Grip strength. ^2^ Sit-to-stand test. ^3^ Appendicular Skeletal Muscle Mass. ^4^ Short Physical Performance Battery. ^5^ Body mass index. ^6^ Waist circumference. ^7^ Total body fat percentage. ^8^ Tricipital skinfold.

**Figure 3:**
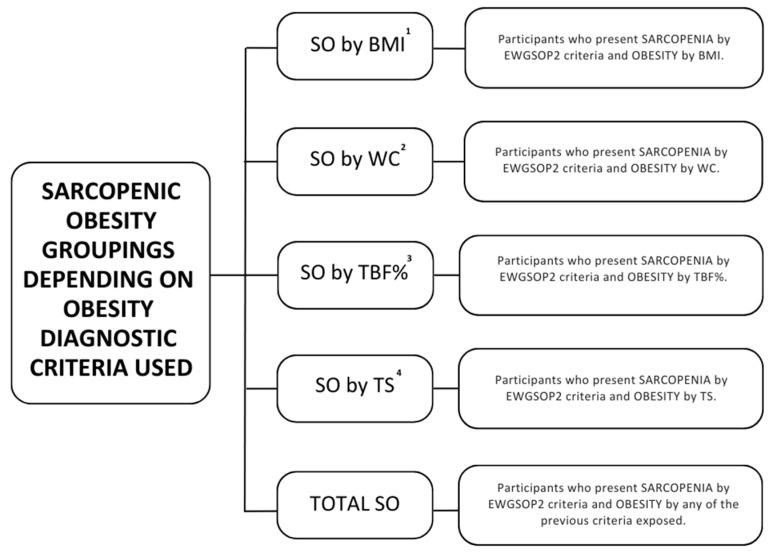
Sarcopenic obesity groupings depending on obesity diagnostic criteria used. ^1^ Body mass index. ^2^ Waist circumference. ^3^ Total body fat percentage. ^4^ Tricipital skinfold.

Finally, a classification of total SO was used to group the subjects who presented sarcopenia and any type of obesity. The complete diagnosis algorithm is reflected in Figure 2. Groupings performed with the aim of assessing SO prevalence based on the obesity criterion selected can be consulted in Figure 3.

### 2.4. Statistical Analysis

Statistical analysis was conducted using IBM SPSS Statistics version 24 for Windows. To assess the normal distribution of the data, the Kolmogorov–Smirnov test was used. Quantitative variables were reported as means and standard deviations, while qualitative data were presented as counts and relative frequencies (percentages).

For comparisons between the two groups, the Student *t*-test was used for normally distributed data, and the Mann–Whitney U test was used for non-normally distributed data. For comparisons involving more than two groups, the ANOVA test was used for normally distributed and homoscedastic data, and the Kruskal–Wallis test was used for non-normally distributed or heteroscedastic data.

Correlations between quantitative indicators were analyzed using Pearson’s Correlation Coefficient (r) (for normally distributed data) and Spearman’s rank correlation coefficient (Spearman Rho) (for non-normally distributed data). Contrasts between qualitative variables were assessed using the Chi-square test, and Fisher’s exact test was used when conditions required it.

A multivariable logistic regression model was used to analyze the association between individual obesity and sarcopenic criteria with the prevalence of sarcopenic obesity. Associations were presented as odds ratios and their corresponding 95% confidence intervals (CIs). The authors identified significant differences with *p*-values ≤ 0.05 in all tests performed.

## 3. Results

The participant group comprised 202 individuals, with 81.2% being women and 18.8% being men. Among women, 61.6% fell within the age range of 65 to 75, while 71% of the 38 male participants were in the same age range.

The proportion of sarcopenia in various stages was similar in both sexes, with 25.6% (95% CI: 18.6–32.6%) in women and 28.9% (95% CI: 13.2–44.7%) in men. Confirmed and severe sarcopenia cases accounted for 7.4% in women and 7.9% in men. The proportion of obesity, based on the criteria under study, was higher in men (97.4% (95% CI: 86.2–99.9%)) than in women (60.4% (95% CI: 52.6–68.2%)). Among women, central obesity was the most common type (53.7%), while in the men’s group, it was attributed to total body fat percentage (TBF%) (55.3%).

For the overall presence of sarcopenic obesity (SO), the women’s group had a proportion of 16.5% (95% CI: 10.5–22.4%), which was lower than the men’s group (29% (95% CI: 13.2–44.7%)). Overall, 26.2% (95% CI: 19.9–32.6%) of participants experienced sarcopenia, 67.3% (95% CI: 60.6–74%) suffered from obesity, and 18.8% (95% CI: 13.2–24.4%) had SO.

The most prevalent type of obesity contributing to SO was central obesity in both sexes (15.2% in women and 29% in men), followed by obesity determined with TBF% (14.6% in women and 15.4% in men). Triceps skinfold criteria were the least influential, showing only 1.2% in women and 6% in men.

Detailed results on sarcopenia, obesity, and SO, based on different diagnostic criteria, age, and sex, can be found in Table 1. Diagnostic criteria significantly impacted the number of subjects diagnosed, with varying results based on age and sex (Table 2).

The proportion of SO did not significantly increase with age in either the women’s group (*p* = 0.520) or the men’s group (*p* = 0.238). No significant difference was observed in the proportion of SO concerning age when the sex of participants was not considered (*p* = 0.267). There were no significant differences in the total proportion of the disease based on sex (*p* = 0.105).

Differences in multiple diagnostic tests for sarcopenia and obesity evaluation among groups with and without SO were significant in the women’s group, except for total Appendicular Skeletal Muscle Mass (ASMM) (*p* = 0.109) and triceps skinfold (*p* = 0.717). In men, significant differences were found only in grip strength (*p* = 0.013) and the sit-to-stand (STS) test (*p* < 0.001).

All values obtained in tests related to SO diagnosis were worse in the group with the disease, except for total ASMM, which was higher in the women’s group with SO and equal between the men’s groups with and without SO.

A statistical model was developed to observe correlations between the main variables used to diagnose obesity and sarcopenia, autonomously. This analysis revealed that the WC diagnostic criterion for obesity was the one that showed the greatest correlation in terms of worse results related to the variables for the diagnosis of sarcopenia. Furthermore, BMI was shown to be the main diagnostic criterion related to obesity with a greater correlation with the rest of the variables related to the disease; thus, a higher degree of BMI resulted in worse values for the rest of the variables related to the obesity study.

In the case of variables related to sarcopenia, the SPPB test was shown to be the most related variable when it was compared with the rest of the diagnostic criteria for the disease; thus, better results in this test resulted in a protective factor regarding the appearance of sarcopenia. The correlations developed for variables related to obesity and sarcopenia studied can be consulted in depth in Table 3.

After identifying the relationships between the main variables that were included in the diagnostic construct of SO, a logistic regression analysis was proposed with the aim of assessing the relationship between each of the diagnostic groupings made by authors for SO and variables related to sarcopenia and obesity on an individualized basis. This analysis showed that the diagnostic grouping of SO determined with BMI was the most relevant in terms of its degree of association between variables related to the two pathologies (sarcopenia and obesity). Hypothetically, SO × BMI presented the greatest number of significant relationships among the variables under study.

On the contrary, diagnostic grouping of SO determined using TS was the one with less clinical relevance in terms of the relationship among the variables under study. The model also showed a positive relationship between variables for the diagnostic groupings of SO × WC and SO × TBF%. All these relationships studied can be consulted in Table 4. 

## 4. Discussion

The methodology used in all revised publications was very heterogeneous. Also, a lack of consensus on SO diagnostic criteria was detected and all of the literature consulted used the latest EWGSOP criteria [16] to assess sarcopenia in subjects. All these mentioned handicaps made it difficult to compare our study with other similar ones. For this reason, we decided to use a general comparison value for sarcopenic obesity obtained using any diagnostic criterion related to obesity. This decision allowed us to less restrictively and more easily compare values in order to contrast them with other publication results. Our study provides insights into the prevalence of sarcopenic obesity (SO) using different diagnostic methods in non-institutionalized and autonomous older adults. The overall SO prevalence, considering any obesity criteria, was 18.8%, which aligns with findings from Gómez-Cabello et al. [6] in Spain with non-institutionalized older individuals (15%). Publications from other countries with participants sharing characteristics with our study subjects reported SO prevalence ranging from 4% to 26% [6,38,39,40,41,42,43]. Considering these comparisons, the prevalence observed in other publications [6,38,39,40,41,42,43,44,45] aligns with the results obtained in our research. In hospital-institutionalized patients, the prevalence of SO ranged between 20% and 30% [46], indicating an increased prevalence compared with the community setting.

Our study found no significant differences in SO prevalence related to age (*p* = 0.267) or sex (*p* = 0.105). Contrary to our findings, publications by Batsis et al. [41,47,48] and Lee et al. [41] suggested an increase in SO prevalence with age, particularly in populations aged 80 years or older. This potential age-related increase might be underestimated in our study due to the limited sample in the 75–85 years group. Regarding sex differences, Kim et al. [10] and Batsis et al. [41,48] observed a higher prevalence in women compared with men. In contrast, Du et al. [40] and one publication by Batsis et al. [47] found a higher prevalence of SO in men. Our study aligns with these varied results, even though statistical significance was not observed between these variables.

In analyzing the values obtained in tests related to sarcopenic obesity (SO) diagnosis, a notable observation is the total amount of Appendicular Skeletal Muscle Mass (ASMM) in both the women and men groups (with and without SO). The total ASMM was found to be higher in the group of women with SO, while in the men’s group, ASMM was the same among those with SO and those without the disease. This circumstance could be attributed to the relationship between muscle mass and participants’ weight or possibly due to a low sample size, particularly in the men’s group. Therefore, it was not concluded that better results for the total ASMM in the SO group contributed to a better overall health status.

Few publications have explored the use of different diagnostic criteria for determining obesity and, concurrently, SO. Moreover, there is limited research attempting to identify differences between these diagnostic criteria or analyze which ones exert the most influence on SO diagnosis. Studies that have found distinctions between diagnostic criteria for SO indicated that the most influential variables were waist circumference and total body fat percentage [4,28]. These findings align with the results of our study.

The logistic regression model carried out by the authors showed that the diagnostic grouping of SO × TS was the least effective determinant for SO diagnosis. According to this study’s result, the researchers suggested the assessment of BMI, WC, and TBF% as diagnostic elements of obesity within the sarcopenic obesity construct. Thus, screening using these three diagnostic criteria for obesity seems to be the most appropriate method due to the high association between these variables and sarcopenic pathology.

Our publication possesses significant methodological strengths. The diagnostic criteria for sarcopenia published in 2019 by the European Working Group on Sarcopenia in Older People (EWGSOP) [16] were rigorously adhered to. Additionally, various prevalence rates of sarcopenic obesity (SO) were presented, considering different diagnostic criteria for obesity. The data were stratified into stable age groups, accompanied by figures that reference all diagnostic tests involved in the assessment of sarcopenia, obesity, and SO.

Despite these strengths, we acknowledge certain limitations in our study, including a non-randomized sample, the study’s single-center nature, and a relatively small sample size in specific age groups and the male cohort. Due to the lack of standardization in the diagnostic criteria for SO, this study highlights the need to establish unification regarding the diagnostic methods used by health professionals around the world to diagnose SO. Furthermore, the prevalence of SO in the community-dwelling population has not been studied in depth, and it is necessary to keep researching this field to obtain data that allows us to delve deeper into the impact of SO in the non-hospitalized population.

We consider that the introduction of the new diagnostic criteria for sarcopenia by the EWGSOP in 2019 [16] makes it necessary to update and review the prevalence of SO under these new criteria. Following the bibliographic claim of some authors [40,43], the standardization of diagnostic criteria is considered relevant, based on international specialized organizations in this field, to unifying the latest academic content on SO.

## 5. Conclusions

In summary, 18.8% of the participants exhibited SO based on some diagnostic criteria related to obesity. Among these criteria, central obesity (17.8%) and total body fat percentage (15.4%) were the most effective in diagnosing this condition, while triceps skinfold yielded a lower prevalence of SO (3%). Despite percentage differences in the prevalence of SO between age groups, no significant age-related differences were found in the men’s group (*p* = 0.238) or the women’s group (*p* = 0.520). Comparing results with data obtained in hospital centers, this study demonstrated a lower prevalence of SO among community-dwelling older adults. This research showed that WC, BMI, and TBF% are strongly related to sarcopenia diagnosis variables, and these variables can be used as a set for the diagnosis of obesity within the SO construct. It is imperative to standardize the criteria for the diagnosis of sarcopenic obesity (SO) through collaboration with international specialized organizations, given the diverse range of academic content published to date.

## Figures and Tables

**Table 1:** Proportions od sarcopenia, obesity, and sarcopenic obesity (SO) in community-dwelling older adults according to age and sex.

	Women			Men			Total N = 202 n (%)
	65–75 y N = 101 n (%)	75–85 y N = 63 n (%)	Total N = 164 n (%)	65–75 y N = 27 n (%)	75–85 y N = 11 n (%)	Total N = 38 n (%)	
*Sarcopenia*							
Without sarcopenia ^1^	80 (79.2)	42 (67)	122 (74.4)	21 (78)	6 (55)	27 (71)	149 (73.8)
Sarcopenia probable ^2^	18 (17.8)	12 (19)	30 (18.3)	5 (19)	3 (27)	8 (21)	38 (18.8)
Sarcopenia confirmed ^3^	2 (2)	4 (6)	6 (3.7)	0 (0)	0 (0)	0 (0)	6 (3)
Sarcopenia severe ^4^	1 (1)	5 (8)	6 (3.7)	1 (4)	2 (18)	3 (8)	9 (4.5)
Total sarcopenia ^5^	21 (20.8)	21 (33)	42 (25.6)	7 (26)	5 (45)	11 (29)	53 (26.2)
*Obesity*							
Without obesity	38 (37.6)	27 (43)	65 (39.6)	1 (4)	0 (0)	1 (3)	66 (32.7)
Obesity per body mass index	45 (44.6)	19 (30)	64 (39)	14 (52)	5 (46)	19 (12)	83 (41.1)
Obesity per central obesity	55 (54.5)	33 (52)	88 (53.7)	26 (96)	11 (100)	37 (97)	125 (61.9)
Obesity per total body fat	39 (38.6)	26 (41)	65 (39.6)	14 (52)	7 (64)	21 (55)	86 (42.6)
Obesity per triceps skinfold	6 (5.9)	3 (5)	9 (5.5)	4 (15)	2 (18)	6 (16)	15 (39.5)
Total obesity	63 (62.4)	36 (57)	99 (60.4)	26 (96)	11 (100)	37 (97)	136 (67.3)
*Sarcopenic Obesity*							
Without sarcopenic obesity ^6^	86 (85.1)	51 (81)	137 (83.5)	21 (78)	6 (55)	27 (71)	164 (81.2)
Sarcopenic obesity per body mass index ^7^	13 (12.9)	7 (11)	20 (12.2)	4 (15)	1 (9)	5 (13)	25 (12.4)
Sarcopenic obesity per central obesity ^8^	15 (14.9)	10 (16)	25 (15.2)	6 (22)	5 (46)	11 (29)	36 (17.8)
Sarcopenic obesity per total body fat ^9^	13 (12.9)	11 (18)	24 (14.6)	4 (15)	3 (37)	7 (18)	31 (15.4)
Sarcopenic obesity per triceps skinfold ^10^	2 (2)	0 (0)	2 (1.2)	3 (11)	1 (9)	4 (11)	6 (3)
Total sarcopenic obesity ^11^	15 (14.9)	12 (19)	27 (16.5)	6 (22)	5 (45)	11 (29)	38 (18.8)

^1^ Muscle mass preserved without any degree of muscle function. ^2^ Reduced muscle strength with preserved muscle quantity or quality and preserved physical performance. ^3^ Reduced muscle strength with low muscle quantity or quality and preserved physical performance. ^4^ Reduced muscle strength with low muscle quantity or quality and low physical performance. ^5^ All cases with any degree of sarcopenic status. ^6^ Without sarcopenia or obesity or with only one of those pathologies. ^7^ Any degree of sarcopenic status with a high value in body mass index. ^8^ Any degree of sarcopenic status with a high value for central obesity. ^9^ Any degree of sarcopenic status with a high percentage of total body fat. ^10^ Any degree of sarcopenic status with a high value for triceps skinfold. ^11^ All cases with any degree of sarcopenic status and obesity per any of the diagnostic criteria used.

**Table 2:** Results of the different tests to assess sarcopenia and obesity according to age, sex, and type of sarcopenic obesity.

	65–75 Years						75–85 Years						WSO (Full Age Range)	SO Total (Full Age Range)
	WSO	SO × BMI	SO × WC	SO × TBF%	SO × TS	SO Total	WSO	SO × BMI	SO × WC	SO × TBF%	SO × TS	SO Total		
Men	N = 21	N = 4	N = 6	N = 4	N = 3	N = 6	N = 6	N = 1	N = 5	N = 3	N = 1	N = 5	N = 27	N = 11
Grip strength (kg)	36.5 ± 7.5	30.5 ± 6.5	31.5 ± 5.5	30.5 ± 6.5	31 ± 5.6	31.5 ± 5.5	32.7 ± 4.5	22 ± 0	28.4 ± 4.4	28.3 ± 6	22 ± 0	28.4 ± 4.4	34.6 ± 6	29.9 ± 2.2
Chair stand test (s)	10.2 ± 2.4	17.5 ± 5.8	17 ± 4.6	17.5 ± 5.8	17.7 ± 2.8	17 ± 4.6	12.6 ± 2.7	20.2 ± 0	17.9 ± 1.6	18.6 ± 1.9	20.2 ± 0	17.9 ± 1.6	11.4 ± 2.6	17.4 ± 0.6
ASMM total (kg)	23.2 ± 3.1	23.3 ± 4.5	23.7 ± 3.5	23.3 ± 4.5	22.8 ± 5.4	23.7 ± 3.5	23 ± 2.5	26.1 ± 0	22.5 ± 3.2	23.9 ± 3.5	26.1 ± 0	22.5 ± 3.2	23.1 ± 2.8	23.1 ± 0.9
Gait speed (m/s)	1.1 ± 0.2	0.9 ± 0.2	0.9 ± 0.2	0.9 ± 0.2	0.9 ± 0.2	0.9 ± 0.2	0.9 ± 0.1	0.6 ± 0	0.9 ± 0.2	0.8 ± 0.3	0.6 ± 0	0.9 ± 0.2	1 ± 0.2	0.9 ± 0
SPPB (score)	10.3 ± 1	8 ± 3	8.3 ± 2	8 ± 3	8.7 ± 3	8.3 ± 2	9.3 ± 2	4 ± 0	5.8 ± 1	5.7 ± 2	4 ± 0	5.8 ± 1	9.8 ± 1.5	7.1 ± 1.8
SARC F test (score)	1.2 ± 1	3 ± 1	2.8 ± 1	3 ± 1	2.3 ± 2	2.8 ± 1	3.2 ± 2	7 ± 0	3.4 ± 3	4.7 ± 3	7 ± 0	3.4 ± 3	2.2 ± 1.5	3.1 ± 0.4
Body mass index (kg/m^2^)	28.1 ± 4.6	33.6 ± 2.3	30.6 ± 5	33.6 ± 2.3	31.8 ± 5.9	30.6 ± 5	28.6 ± 4.1	33.3 ± 0	26.4 ± 4	28.3 ± 4.3	33.3 ± 0	26.4 ± 4	28.4 ± 4.4	28.5 ± 3
Waist circumference (cm)	101.4 ± 9.6	109.8 ± 3.8	104.8 ± 8.7	109.8 ± 3.8	105.7 ± 5.6	104.8 ± 8.7	104.4 ± 8.2	109.3 ± 0	104.2 ± 3.8	104 ± 4.7	109.3 ± 0	104.2 ± 3.8	102.9 ± 8.9	104.5 ± 0.4
Total body fat (%)	26.3 ± 6.1	32.7 ± 3.7	29.2 ± 6.3	32.7 ± 3.7	31.3 ± 6.7	29.2 ± 6.3	28.9 ± 4.1	37.4 ± 0	28.8 ± 5.6	32 ± 4.8	37.4 ± 0	28.8 ± 5.6	27.6 ± 5.1	29 ± 0.3
Triceps skinfold (mm)	12 ± 5.1	19 ± 9.5	18.5 ± 8.2	19 ± 9.5	25.7 ± 3.1	18.5 ± 8.2	15 ± 4.1	27 ± 0	14.2 ± 7.9	16.7 ± 10	27 ± 0	14.2 ± 7.9	13.5 ± 4.6	16.4 ± 3
Women	N = 86	N = 13	N = 15	N = 13	N = 2	N = 15	N = 51	N = 7	N = 10	N = 11	N = 0	N = 12	N = 137	N = 27
Grip strength (kg)	21.5 ± 4.1	16.2 ± 6.7	16 ± 6.2	16.6 ± 6.3	13 ± 1.4	16 ± 6.2	19 ± 4.3	14.3 ± 1.5	14.7 ± 2.3	14.8 ± 2.2	-	14.9 ± 2.1	20.3 ± 4.2	15.5 ± 0.8
Chair stand test (s)	10.6 ± 2.4	14.1 ± 4.6	14.2 ± 4.6	14.6 ± 4.8	12.8 ± 6.7	14.2 ± 4.6	11.8 ± 2.9	15.1 ± 5.8	16.2 ± 5.3	16.3 ± 5.3	-	16.2 ± 5.1	11.2 ± 2.7	15.2 ± 1.4
ASMM total (kg)	16.8 ± 2.4	18.2 ± 1.7	17.6 ± 2.3	18 ± 2	16.5 ± 1.9	17.6 ± 2.3	15.7 ± 2.4	17.8 ± 3.3	17 ± 3	17.1 ± 2.8	-	16.8 ± 2.8	16.3 ± 2.4	17.2 ± 0.6
Gait speed (m/s)	1.1 ± 0.2	0.9 ± 0.2	0.9 ± 0.2	0.9 ± 0.2	1 ± 0.3	0.9 ± 0.2	1 ± 0.2	0.7 ± 0.2	0.7 ± 0.2	0.7 ± 0.2	-	0.7 ± 0.2	1.1 ± 0.2	0.8 ± 0.1
SPPB (score)	10.3 ± 2	8.7 ± 2	8.5 ± 2	8.5 ± 2	8.5 ± 2	8.5 ± 2	9.6 ± 2	8.4 ± 2	8.5 ± 2	8.2 ± 2	-	8.3 ± 2	9.9 ± 2	8.4 ± 0.1
SARC F test (score)	1.7 ± 1	3.3 ± 2	3.3 ± 1	3.1 ± 1	4 ± 0	3.3 ± 1	2.7 ± 1	3.9 ± 0	4.1 ± 1	4.1 ± 1	-	4 ± 1	2.2 ± 1	3.7 ± 0.5
Body mass index (kg/m^2^)	27.4 ± 4.4	30.7 ± 2	30.1 ± 2.6	30.5 ± 2.3	31.4 ± 3	30.1 ± 2.6	25.9 ± 3.7	32 ± 2.9	30 ± 4	30 ± 3.7	-	29.6 ± 3.7	26.7 ± 4.1	29.9 ± 0.4
Waist circumference (cm)	89.1 ± 9.5	97.8 ± 5.1	97.7 ± 4.7	97.8 ± 5.1	97.8 ± 7.4	97.7 ± 4.7	87.6 ± 8.2	100.4 ± 10.8	98.9 ± 8.5	97.3 ± 9.6	-	96.6 ± 9.5	88.4 ± 8.9	97.2 ± 0.8
Total body fat (%)	35.9 ± 5.2	41.4 ± 2.9	40.9 ± 3.1	41.7 ± 2.6	41 ± 1.8	40.9 ± 3.1	35.9 ± 5.3	43.5 ± 3.7	42.2 ± 4	42.5 ± 3.4	-	41.9 ± 3.7	25.9 ± 5.3	41.4 ± 0.7
Triceps skinfold (mm)	20.9 ± 5.5	22.5 ± 6.3	22.5 ± 5.9	23 ± 6.2	28 ± 11.3	22.5 ± 5.9	20.3 ± 5.4	19.1 ± 3.6	19.4 ± 4.1	19.3 ± 4	-	19.3 ± 3.8	20.6 ± 5.5	20.9 ± 2.3

Values are presented as mean ± standard deviation. WSO: without sarcopenic obesity. SO: sarcopenic obesity. BMI: body mass index. WC: waist circumference. TBF%: total body fat percentage. TS: triceps skinfold. ASMM: Appendicular Skeletal Muscle Mass. SPPB: Short Physical Performance Battery.

**Table 3:** Associations between obesity and sarcopenia diagnosis variables.

		Obesity Diagnosis Variables				Sarcopenia Diagnosis Variables				
		BMI ^1^	WC ^2^	TS ^3^	TBF% ^4^	CST Test ^5^	Grip Strength	ASSM ^6^	Gait Speed	SPPB Test ^7^
Obesity diagnosis variables	BMI ^1^	1	0.718 **	0.396 **	0.825 **	0.135	0.052	0.676 **	−0.131	−0.153 *
	WC ^2^	0.718 **	1	0.037	0.573 **	0.241 **	0.314 **	0.719 **	−0.143 *	−0.222 **
	TS ^3^	0.396 **	0.037	1	0.652 **	0.068	−0.255 **	0.005	−0.129	−0.042
	TBF% ^4^	0.825 **	0.573 **	0.652 **	1	0.146 *	0.054	0.451 **	−0.072	−0.085
Sarcopenia diagnosis variables	CS test ^5^	0.135	0.241 **	0.068	0.146 *	1	−0.104	0.134	−0.491 **	−0.709 **
	Grip strength	0.052	0.314 **	−0.255 **	0.054	−0.104	1	0.353 **	0.225 **	0.161 *
	ASSM ^6^	0.676 **	0.719 **	0.005	0.451 **	0.134	0.353 **	1	−0.104	−0.183 **
	Gait Speed	−0.131	−0.143 *	−0.129	−0.072	−0.491 **	0.225 **	−0.104	1	0.503 **
	SPPB test ^7^	−0.153 *	−0.222 **	−0.042	−0.085	−0.071 **	0.161 *	−0.183 **	0.503 **	1

Values presented as Rho (Spearman correlation test). * *p <* 0.05 (bilateral). ** *p <* 0.01 (bilateral). ^1^ Body mass index. ^2^ Waist circumference. ^3^ Triceps skinfold. ^4^ Total body at. ^5^ Chair stand test. ^6^ Appendicular Skeletal Muscle Mass. ^7^ Short Physical Performance Battery test.

**Table 4:** Logistic regression model for different SO groupings and principal obesity and sarcopenia variables.

	SO × BMI		SO × WC		SO × TBF%		SO × TS		SO Total	
	OR (95% CI)	*p*	OR (95% CI)	*p*	OR (95% CI)	*p*	OR (95% CI)	*p*	OR (95% CI)	*p*
Body mass index	1.50 (1.20 to 1.87)	<0.001							1.20 (1.01 to 1.42)	0.037
Waist circumference	10.41 (1.01 to 107.6)	0.049	12.9 (3.0 to 55.5)	<0.001	11.4 (1.73 to 75.0)	0.01			10.2 (2.34 to 44.6)	0.002
Triceps skinfold	0.87 (0.77 to 0.98)	0.025					1.25 (1.06 to 1.48)	0.007		
Total body fat					1.06 (0.95 to 1.19)	0.27			0.88 (0.77 to 0.99)	0.04
ASMM total			1.84 (1.04 to 3.25)	0.048	2.42 (1.25 to 4.68)	0.009				
Grip strength	0.829 (0.74 to 1.15)	<0.001	0.89 (0.82 to 0.96)	0.001	0.86 (0.77 to 0.94)	0.002			0.87 (0.79 to 0.95)	0.001
Chair stand test	1.35 (1.15 to 1.59)	<0.001	1.51 (1.29 to 1.77)	<0.001	1.54 (1.29 to 1.83)	<0.001	1.39 (1.11 to 1.74)	0.004	1.57 (1.33 to 1.85)	<0.001
Gait speed										
SPPB										

Values presented as odds ratio per unit (95% confidence interval) (OR (95% CI). SO: sarcopenic obesity. BMI: body mass index. WC: waist circumference. TBF%: total body fat percentage. TS: triceps skinfold. ASMM: Appendicular Skeletal Muscle Mass. SPPB: Short Physical Performance Battery.

## Data Availability

The data presented in this study are available on request from the corresponding author.
